# Fe_3_O_4_@chitosan-tannic acid bionanocomposite as a novel nanocatalyst for the synthesis of pyranopyrazoles

**DOI:** 10.1038/s41598-021-99121-2

**Published:** 2021-10-08

**Authors:** Maryam Kamalzare, Mohammad Reza Ahghari, Mohammad Bayat, Ali Maleki

**Affiliations:** 1 Department of Chemistry, Faculty of Science, Imam Khomeini International University, Qazvin, Iran; 2 Catalysts and Organic Synthesis Research Laboratory, Department of Chemistry, Iran University of Science and Technology, Tehran, 16846-13114 Iran

**Keywords:** Chemistry, Catalysis, Heterogeneous catalysis

## Abstract

Recently magnetic nanocatalyst has attracted considerable attention because of its unique properties, including high performance, easy separation from the reaction mixture, and recyclability. In this study, a novel magnetic bionanocomposite was synthesized with chitosan and tannic acid as a natural material. The synthesized bionanocatalyst was characterized by essential analysis. Fe_3_O_4_@chitosan-tannic acid as a heterogeneous nanocatalyst was successfully applied to synthesize pyranopyrazole and its derivatives by a one-pot four-component reaction of malononitrile, ethyl acetoacetate, hydrazine hydrate, and various aromatic aldehyde. At the end of the reaction, the nanocatalyst was separated from the reaction mixture and was reused several times with no significant decrease in its catalytic performance. Simple purification of products, the ability for recovering and reusing the nanocatalyst, eco-friendliness, high yields of pure products, mild reaction conditions, short reaction time, non-toxicity, economically affordable are some of the advantages of using the fabricated nanocatalyst in the synthesis of pyranopyrazole.

## Introduction

**Figure 1:**
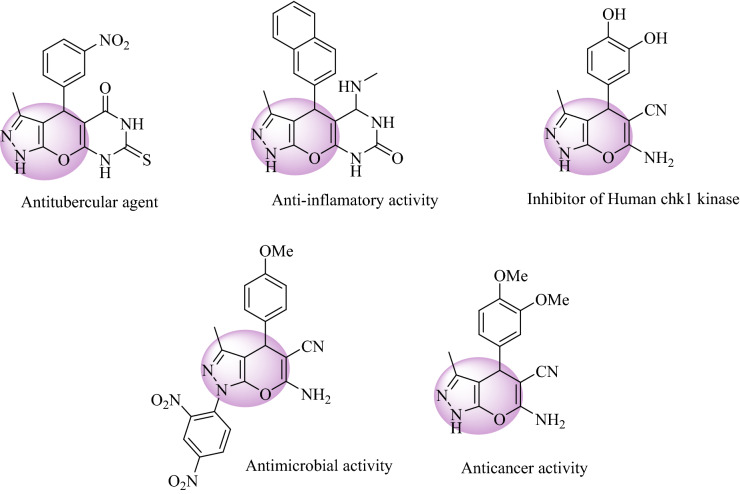
Selected examples of pyranopyrazole derivatives with pharmaceutical and biological activity.

One of the best ways to synthesize heterocyclic compounds starting from simple and readily available materials is multicomponent reactions (MCRs). MCRs present high efficiency due to the depreciation of the synthesis steps to form complex compounds and are time-saving and atom economy. In this regard, designing new MCRs associated with principles of green chemistry has attracted considerable attention^1,2^. Pyranopyrazole, which could be synthesized through a four-component reaction, is one of the most important types of oxygen–nitrogen heterocyclic compounds with lots of biological, pharmaceutical, and agrochemical applications. For instance, antimicrobial, antiplatelet, anti-inflammatory, antitumor, antibacterial, insecticidal, fungicidal, antitubercular agent, herbicidal, an inhibitor of human chk1 kinase, as well as UV absorber are some of the important activities of pyranopyrazole and its derivatives (Fig. 1). Because of the great importance and numerous utilizations of pyranopyrazole and its derivatives, modifying the procedure for its synthesis are at the top of scientific research^3,4^. Using heterogeneous nanocatalyst to synthesize pyranopyrazole has attracted great importance among previous methods on account of high efficiency. For instance, poly(ethylene imine)-modified magnetic halloysite nanotubes as a novel, efficient and recyclable catalyst for the synthesis of dihydropyrano[2,3-c]pyrazole derivatives; green reaction condition, easy workup, an excellent yield of the products, easy isolation of the catalyst by an external magnet and the ability to recovering and recycling the catalyst without any further decrease in catalytic activity are the main advantages of this method^5^. Another efficient and green heterogeneous catalyst for the synthesis of pyranopyrazole and its derivatives is Ru^III^@CMC/Fe_3_O_4_; high performance, short time, mild reaction condition, easy workup, reusability, simple preparation of the catalyst with an external magnet, and green catalytic procedure are the main advantages of this procedure^6^.

**Figure 2:**
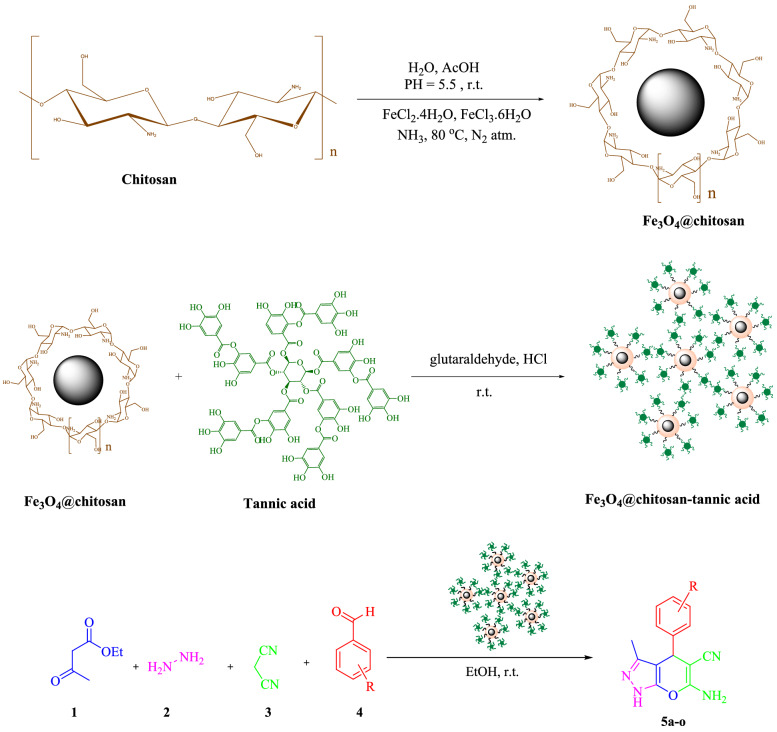
Synthesis of Fe_3_O_4_@chitosan-tannic acid bionanocomposite and its catalytic activity in the synthesis of pyranopyrazole and its derivatives.

Fe_3_O_4_@SiO_2_@PTS-DABA^7^, nano-Al_2_O_3_/BF_3_/Fe_3_O_4_^8^, CoCuFe_2_O_4_^9^, CMCSO_3_H^10^, Fe_3_O_4_/GO@melamine-ZnO Nanocomposite^11^, SnO_2_ QDs^12^, amberlyst A21^13^ are another catalytic system for the synthesis of pyranopyrazole and its derivatives. One fundamental and beneficial way to sketch new green MCRs is to develop and apply unique and particular catalysts for the synthesis of heterocyclic compounds. The most critical aspects of homogeneous catalysts are their high catalytic activity, but the workup process, especially the separation of the catalyst from the reaction mixture, is challenging. Therefore, the recyclability and reusability of the catalyst is the main problem, and a large amount of catalyst will waste. Additionally, even after a great and practical separation of homogeneous catalyst from the reaction mixture, a small amount of catalyst has remained in the final product, which is troublemaking, especially in drug industries. Versus heterogeneous catalysts could easily and quickly be separated from the reaction mixture and reused without loss in their amount during the purification process. The most obvious limitation in this area is the low activity of the heterogeneous catalyst; this limitation prevented an extensive application in industrial usage. Therefore, researchers have spotlighted introducing novel catalysts with both advantages of homogeneous and heterogeneous catalysts^14–16^. For this purpose, designing and synthesizing heterogeneous nanocatalysts is a fundamental way to achieve catalysts with special properties. Synthesizing nanoscale catalysts is one of the best ways to achieve high activity for the heterogeneous catalytic system. Considering that the increasing surface-to-volume ratio of the nanoscale heterogeneous catalyst leads to a unique catalyst with the ability of easy separation and high performance^17,18^. In consideration of easy separation from the reaction mixture because of magnetic properties, high active surface area, non-toxicity, the ability to functionalization on the surface, magnetic nanoparticles are at the highest point of attention for using them as the catalyst. Despite all of these substantial properties, magnetic nanoparticles aggregate to large clusters that cause limited dispersion in the mixture of the reaction following a decrease in the catalytic efficiency. Accordingly, coating magnetic nanoparticles with some materials prevents aggregation of magnetic nanoparticles. Natural materials are the best option for coating magnetic nanoparticles and lead to green nanoscale composite as a novel catalyst for various organic reactions^19–22^. Chitosan is one of the most abundant natural polymers derived from the deacetylation of chitin. Chitosan is a natural cationic biopolymer with good solubility in a dilute acidic medium with the protonation of the NH_2_ group. Chitosan is appropriate for designing nanocomposites owing to its remarkable biodegradability, non-toxicity, bioactivity, low cost, and readily available. Considering that chitosan has lots of -NH_2_ and -OH groups, it could react easily with other materials to fabricate new functionalized composite^23^. Tannic acid is a polyphenol with weak acidity and is a special form of tannins. Tannic acid can be found in various plant tissue. Owing to the fact that tannic acid is consist of phenolic groups, it has a good ability for manufacturing heterogeneous catalyst. Low cost, availability, biodegradability, and biocompatibility are some of the properties of tannic acid which persuade researchers to use it^24^. To continue our research in designing heterogeneous magnetic nanocatalyst in the direction of the principle of green chemistry^24–30^, herein, we wish to report a novel procedure for the synthesis of pyranopyrazole and its derivatives with Fe_3_O_4_@chitosan-tannic acid as a bionanocatalyst (Fig. 2). From the standpoint of green chemistry presence of chitosan and tannic acid in the nanocatalyst enhances the biocompatibility of the method and makes the process interesting for industrial usages. Besides, due to the existence of magnetic nanoparticles, the bionanocatalyst could be separated from the reaction mixture easily with an external magnet; also, because of the chemical stability of the bionanocatalyst, it could be reused multiple times with almost consistent efficiency. Herein, we report a reaction for the synthesis of pyrano[2,3-c]pyrazole and its derivatives via a four-component reaction of hydrazine hydrate, ethyl acetoacetate, malononitrile, and various aromatic aldehyde in ethanol as a green solvent.

## Experimental

### General

The solvents, chemicals, and reagents were purchased from various commercial companies such as Merck, Sigma-Aldrich, and Fluka and were used as received. Analytical thin-layer chromatography (TLC) was performed using Merck silica gel GF254 plates. IR spectra were measured with a Shimadzu IR-470 spectrometer. The NMR spectra were recorded with a Bruker DRX-300 AVANCE instrument (300 MHz for 1H and 75.4 MHz for 13C). X-ray diffraction (XRD) patterns of the solid powders were recorded with a JEOL JDX-8030 (30 kV, 20 mA). Thermal analysis was taken by Bahr-STA 504 instrument under the air atmosphere. Morphological investigations were studied by field-emission scanning electron microscopy (FE-SEM, MIRA 3 TESCAN). EDX analysis was recorded on Numerix DXP-X10P. The transmission electron microscopy (TEM) was provided on a Philips CM200.

### Preparation of Fe_3_O_4_@chitosan-tannic acid bionanocomposite

#### *Synthesis of Fe*_*3*_*O*_*4*_*@chitosan bionanocomposite*

At the first stage, 0.5 g chitosan was dissolved in dilute acidic deionized water (pH 5.5 by acetic acid) at 80 °C under N_2_ atmosphere. Then two aqueous solutions of FeCl_3_.6H_2_O and FeCl_2_.4H_2_O with the molar ratio of 2:1 were added to the chitosan solution. In the following concentrate, NH_3_ was added to the mixture until pH 12 was obtained. The color of the solution changed to black, and Fe_3_O_4_@chitosan composite was synthesized via an in situ process. Fe_3_O_4_@chitosan was collected by an external magnet, washed multiple times with deionized water, and dried in the oven at 80 °C.

#### Synthesis of Fe_3_O_4_@chitosan-tannic acid bionanocomposite

At the second stage, 0.4 g tannic acid dissolved in 10 ml of deionized water and simultaneously 0.06 g Fe_3_O_4_@chitosan dispersed in 15 ml deionized water via ultrasonication for 10 min. The tannic acid solution was added to the aqueous mixture of Fe_3_O_4_@chitosan, and the mixture was stirred for 15 min at room temperature. After that, 1 ml HCl (1 M) and 1 ml glutaraldehyde were added to the mixture and were stirred for 20 min. Finally, the synthesized Fe_3_O_4_@chitosan-tannic acid bionanocomposite was separated with an external magnet, washed with deionized water, and dried at room temperature.

#### General procedure for the synthesis pyrano[2,3-c]pyrazole via Fe_3_O_4_@chitosan-tannic acid as heterogeneous nanocatalyst

Fe_3_O_4_@chitosan-tannic acid (0.02 g) was added to the mixture of malononitrile (1 mmol), ethyl acetoacetate (1 mmol), hydrazine hydrate (1 mmol), and various aldehyde (1 mmol) in 3 ml ethanol as a solvent and was stirred at room temperature for an appropriate time to synthesize various derivatives of pyranopyrazole. After the reaction was completed (TLC monitoring, eluent: n-hexane:ethyl acetate 3:1), the nanocatalyst was separated from the reaction mixture by an external magnet, and the pure product was achieved. In some cases, the recrystallization from ethanol gave rise to the desired product in pure form.

#### Spectral data for selected products

6-Amino-3-methyl-4-(*p*-tolyl)-2,4-dihydropyrano[2,3-c]pyrazole-5-carbonitrile (5 g). Milky white solid. ^1^HNMR (DMSO-d_6_, 300 MHz), *δ* (ppm): 1.76 (s, 3H), 2.25 (s, 3H), 4.52 (s, 1H), 6.83 (s, 2H, NH_2_), 7.28 (d, 2H, j = 7 Hz), 7.76 (d, 2H, j = 7 Hz), 12.06 (s, 1H, NH). ^13^C NMR (DMSO-d6, 75 MHz): δ (ppm): 10.4, 20.9, 21.3, 57.9, 109.8, 119.13, 127.5, 129.2, 130.5, 131.8, 133.8, 164.4, 179.7.

6-Amino-3-methyl-4-(2-nitrophenyl)-2,4-dihydropyrano[2,3-c]pyrazole-5-carbonitrile (*5c*). Pale yellow solid. ^1^HNMR (DMSO-d_6_, 300 MHz), *δ* (ppm): 1.74 (s, 3H), 5.08 (s, 1H), 7.03 (s, 2H, NH_2_), 7.31–7.87 (4H, Ar), 12.19 (s, 1H, NH).

## Results and discussion

### Characterization of Fe_3_O_4_@chitosan-tannic acid bionanocomposite

**Figure 3:**
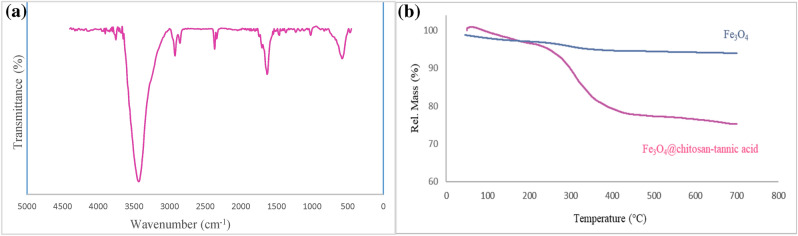
(**a**) FT-IR spectrum of Fe_3_O_4_@chitosan-tannic acid bionanocomposite and (**b**) TGA curves of Fe_3_O_4_@chitosan-tannic acid bionanocomposite and Fe_3_O_4_ nanoparticles.

The structure and morphology of bionanocomposite were investigated by various conventional instrumental techniques. The FT-IR spectrum of Fe_3_O_4_@chitosan-tannic acid is presented in Fig. 3a. In the spectrum of Fe_3_O_4_@chitosan-tannic acid, a broad absorption band between 3600–2900 cm^−1^ shows the presence of numerous hydroxyl groups. The peak in 1632 cm^-1^ is related to the stretching vibration of carbonyl groups. The peak at 578 cm^−1^ represented the Fe–O groups in the bionanocomposite.

The thermal resistance of the synthesized Fe_3_O_4_@chitosan-tannic acid has been investigated by thermogravimetric analysis (TGA), as illustrated in Fig. 3b. TGA analysis was performed under air atmosphere and in the range of 50–800 °C. At the beginning of the graph, there is a little increase in the weight of the bionanocomposite due to the physical absorption of moisture of the air. From the range of 100–220 °C, there is a decrease in the weight of the bionanocomposite pertaining to the water and other molecules that are physically absorbed on the surface of the bionanocomposite. In this stage, the weight percentage was reduced to ca. 96%. The first significant degradation is at 220–450 °C due to the decomposition of the organic structure of bionanocomposite. Also, by examining the thermal analysis diagram related to Fe_3_O_4_ at the same range, it is clear that only 6.5% of its mass is reduced, indicating water molecules and impurities that have been adsorbed on the surface of iron oxide nanoparticles.

**Figure 4:**
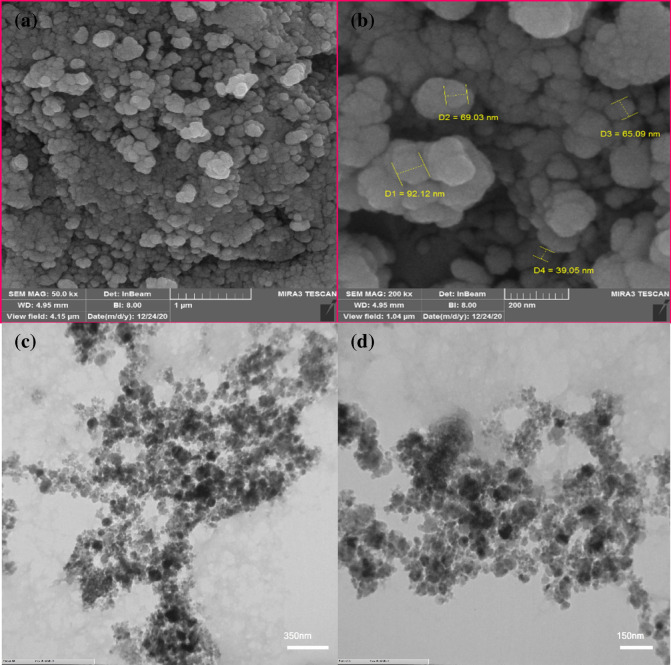
(**a,b**) SEM images of Fe_3_O_4_@chitosan-tannic acid bionanocomposite and (**c,d**) TEM images of Fe_3_O_4_@chitosan-tannic acid bionanocomposite.

Field emission electron microscopy (FE-SEM) image was used to indicate the shape and the size of the bionanocomposite. As seen in Fig. 4a, the appropriate distribution of the spherical Fe_3_O_4_ nanoparticles is presented on the chitosan surface.

To obtain more confirmation about the structure TEM image of the bionanocomposite was investigated. As seen in Fig. 4b, the dark spots are iron oxide nanoparticles covered by the organic part of the bionanocomposite. From the TEM image, the average size of the Fe_3_O_4_ NPs is determined in ca. 20 nm.

**Figure 5:**
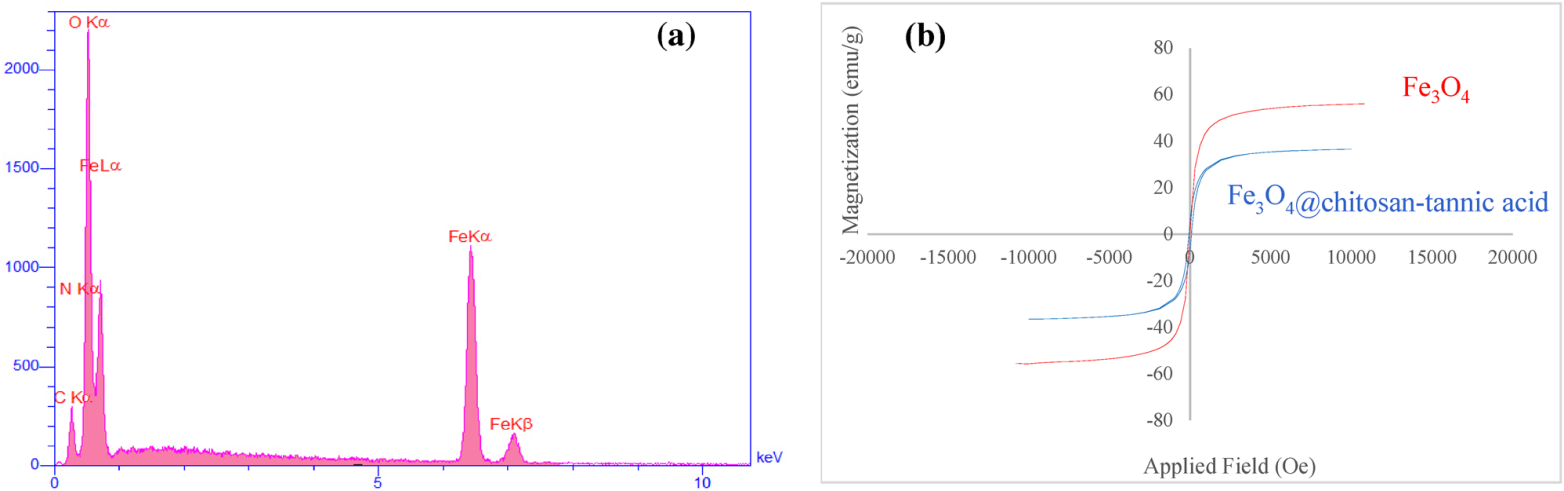
(**a**) EDX analysis of Fe_3_O_4_@chitosan-tannic acid bionanocomposite and (**b**) VSM magnetization curve of Fe_3_O_4_ and Fe_3_O_4_@chitosan-tannic acid bionanocomposite.

EDX analysis was done for investigation of the presence of elements in the structure of bionanocomposite (Fig. 5a). EDX analysis show that bionanocomposite consists of C, O, N, and Fe atoms. Besides, the elemental mapping of EDX patterns shows the presence of C, O, N, and Fe elements in the bionanocomposite (see Figs S1 in Supporting Information File).

VSM vibrating sample magnetometer is a method for study the magnetic property of synthesized bionanocomposite. Figure 5b shows the hysteresis loops of the magnetite nanoparticles (Fe_3_O_4_), and Fe_3_O_4_@chitosan-tannic acid and saturation magnetization (Ms), remanence magnetization (Mr), and coercive force (Hc) data are summarized in Table S1 in Supplementary Information File. Data indicate that Fe_3_O_4_, Fe_3_O_4_@chitosan-tannic acid have superparamagnetic behavior as evidenced by low Hc and Mr on the magnetization loop. The corresponding saturation magnetization (Ms) for Fe_3_O_4_ nanoparticles is 56.07 emu/g, whereas the magnetization of Fe_3_O_4_@chitosan-tannic acid has been saturated in 36.54 emu/g.

**Figure 6:**
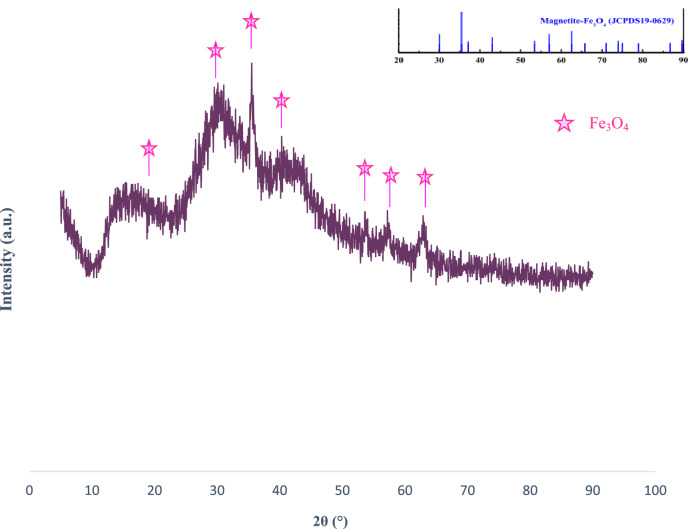
The XRD pattern of Fe_3_O_4_@chitosan-tannic acid bionanocomposite.

The XRD pattern of bionanocomposite is shown in Fig. 6. As can be seen, the obtained diffraction pattern has been compared with the references pattern of Fe_3_O_4_ nanoparticles. Fe_3_O_4_@chitosan-tannic acid represented the main peaks with dispersion angle 2θ = 30.26, 35.66, 43.32, 53.52, 57.41, 62.98, which were consistent with the characteristic peaks of Fe_3_O_4_ (JCPDS No. 19-0629). Besides using the Scherrer equation (D = *k*λ/*β* cos *θ*), the average crystallite size of the particles was calculated (D = 13 nm).

### Catalytic activity of Fe_3_O_4_@chitosan-tannic acid bionanocomposite

#### Optimization of the reaction conditions & catalytic application in the synthesis of pyranopyrazole and its derivatives

In order to monitor the catalytic performance of the synthesized bionanocomposite, the reaction conditions were initially optimized by using various catalytic ratios in different solvents and temperatures, for the synthesis of product 5a, by using hydrazine hydrate, ethyl acetoacetate, malononitrile, and 4-chlorobenzaldehyde as reported in Table S2 in Supplementary Information File. As reported in Table S2 the best result was obtained with 0.02 g nanocatalyst in ethanol as a solvent and at room temperature. The reaction was monitored with thin layer chromatography (TLC). At the end of the reaction, the nanocatalyst was separated from the reaction mixture by an external magnet, and pure products were achieved. In some cases, the product is recrystallized with hot ethanol.

In addition, to reveal the generality and evaluate the scope of this method, the optimized reaction condition was used to synthesize different derivatives of pyranopyrazole. As can be seen in Table 1, aldehyde with both electron-withdrawing groups and electron-donating groups were reacted well and produced the pure products in high yields.

**Table 1:** Synthesis of pyranopyrazole derivatives (**5a–o**) via one-pot four-component reaction of ethyl acetoacetate, hydrazine hydrate, malononitrile, and various aldehydes, catalyzed by Fe_3_O_4_@chitosan-tannic acid in ethanol at room temperature.

Entry	Aldehyde	Product	Time (min)	Isolated yield (%)	m.p. (obsd.) (°C)	m.p. (lit.) (°C)
1	4-Chlorobenzaldehyde	**5a**	15	94	230–232	231–233^31^
2	4-Nitrobenzaldehyde	**5b**	15	95	240–242	239–242^8^
3	2-Nitrobenzaldehyde	**5c**	20	92	223–225	220–222^32^
4	2,4-Dichlorobenzaldehyde	**5d**	25	90	228–230	229–230^33^
5	4-Fluorobenzaldehyde	**5e**	20	94	171–173	168–170^34^
6	3-Bromobenzaldehyde	**5f**	30	90	183–185	177–179^35^
7	4-Methylbenzaldehyde	**5g**	30	94	190	192–194^36^
8	4-Methoxybenzaldehyde	**5h**	25	93	165–167	168–170^37^
9	4-Hydroxybenzaldehyde	**5i**	30	89	215	220–222^38^
10	4-(Dimethylamino)benzaldehyde	**5j**	25	86	210	211–213^39^
11	3-Hydroxybenzaldehyde	**5k**	35	91	215–217	220–222^38^
12	2,4-Dimethoxybenzaldehyde	**5l**	40	88	220–222	222–224^40^
13	5-Bromosalicylaldehyde	**5m**	30	89	215–217	212–214^41^
14	3-Methoxysalicylaldehyde	**5n**	45	90	236–238	240–242^42^
15	3,4,5-Trimethoxybenzaldehyde	**5o**	30	92	187–189	194–196^43^

#### Catalyst reusability

One of the most important and practical aspects of Fe_3_O_4_@chitosan-tannic acid as a catalyst is recyclability. In the direction of investigating the recyclability of bionanocatalyst, the model reaction was monitored for this purpose. After completing the reaction, the nanocatalyst was separated from the reaction mixture and was washed multiple times with ethanol, and dried at room temperature. Consequently, the nanocatalyst could be reused seven times without any considerable decrease in its catalytic activity (see Figs S2 in Supporting Information File). Besides, the stability of the recycled catalyst was identified by XRD pattern and EDX analysis. It was revealed that no significant changes occurred in the structure of the recycled Fe_3_O_4_@chitosan-tannic acid. As can be observed in the XRD pattern, all of the distinct indicative peaks appeared in both XRD patterns. EDX analysis of the obtained catalyst after seven cycles in the reaction was presented, and there was no considerable change in the EDX analysis (see Figs S3 and S4 in Supporting Information File).

### Proposed mechanism

**Figure 7:**
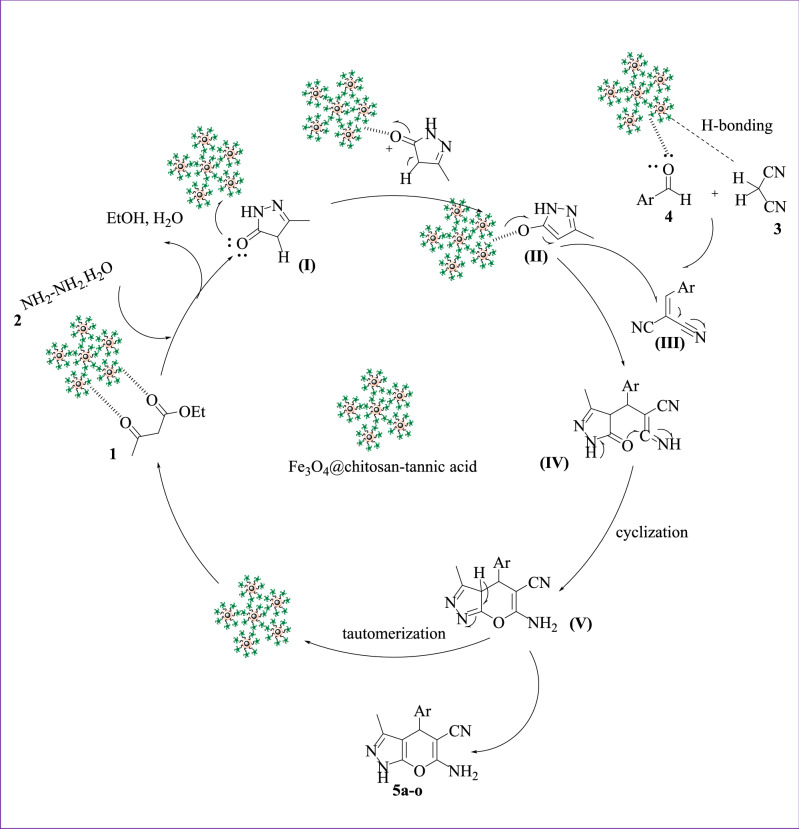
A plausible mechanism for the one-pot four-component reaction of ethyl acetoacetate **(1)** hydrazine hydrate **(2)** malononitrile **(3)** and various aldehydes **(4)**, catalyzed by Fe_3_O_4_@chitosan-tannic acid in ethanol at room temperature.

A proposed mechanism for the reaction is represented in Fig. 7. Bionanocomposite plays a vital role in the formation of pure pyranopyrazole derivatives. As can be seen, the bionanocomposite activate the carbonyl groups of ethyl acetoacetate, and by the nucleophilic attack of hydrazine upon the carbonyl group of ethyl acetoacetate and after losing of H_2_O, another group of hydrazine attack to another carbonyl group of ethyl acetoacetate and removal of ethanol, the pyrazolone ring (I) was formed. After the formation of pyrazolone ring (I), bionanocatalyst will receive a pair of electrons on the oxygen atom of the carbonyl group and then give enol form of pyrazolone ring (II). The intermediate (III) was formed due to the knoevenagel condensation of aldehyde and malononitrile promoted by activating the carbonyl group of aldehyde and activating methylene group of malononitrile with H-bonding. In the following, Michael addition between intermediate (II) and (III) gave intermediate (IV), which undergoes intramolecular cyclization (intermediate V). Finally, the tautomerization of intermediate (V) gave the desired products (5a–o).

## Conclusion

In a brief explanation, a new heterogeneous magnetic nanocatalyst synthesized by chitosan, tannic acid and the structural features of the bionanocomposite were investigated by FT-IR, FE-SEM, VSM, TEM, XRD, EDX, and TGA analysis. Fe_3_O_4_@chitosan-tannic acid synthesized by an easy procedure and low-cost materials. A partial amount of the fabricated bionanocomposite (0.02 g) was adequate for the synthesis of pyranopyrazole and its derivatives in ethanol as a green solvent and a short reaction time with resulting high yields of pure products. Pyranopyrazole is one of the most important heterocyclic compounds, which has lots of pharmaceuticals and biological activity. In this study, we reported an effective and practical method for the synthesis of pyranopyrazole and its derivatives. Biodegradability is one of the most important aspects of the synthesized nanocatalyst, which could be due to the natural components, including chitosan and tannic acid. The great magnetic behavior of nanocatalyst, which origins from the Fe_3_O_4_ nanoparticles fabricated via in situ procedure, is cause to easily separation of nanocatalyst from the reaction mixture by an external magnet. This new procedure for the synthesis of pyranopyrazole and its derivatives is in accordance with the principle of sustainable and green chemistry and could be scaled up.

## Supplementary Information

Supplementary Information.

## References

[1] Afshari R, Shaabani A (2018). Materials functionalization with multicomponent reactions: State of the art. ACS Comb. Sci..

[2] Dömling A, Wang W, Wang K (2012). Chemistry and biology of multicomponent reactions. Chem. Rev..

[3] Mamaghani M, Hossein Nia R (2019). A review on the recent multicomponent synthesis of pyranopyrazoles. Polycycl. Aromat. Compd..

[4] Koohshari M, Dabiri M, Salehi P (2014). Catalyst-free domino reaction in water/ethanol: An efficient, regio- and chemoselective one-pot multicomponent synthesis of pyranopyrazole derivatives. RSC Adv..

[5] Hajizadeh Z, Maleki A (2018). Poly(ethylene imine)-modified magnetic halloysite nanotubes: A novel, efficient and recyclable catalyst for the synthesis of dihydropyrano[2,3-c]pyrazole derivatives. Mol. Catal..

[6] Chen Y, Zhang Z, Jiang W, Zhang M, Li Y (2018). RuIII@CMC/Fe_3_O_4_ hybrid: An efficient, magnetic, retrievable, self-organized nanocatalyst for green synthesis of pyranopyrazole and polyhydroquinoline derivatives. Mol. Divers..

[7] Karami S, Dekamin MG, Valiey E, Shakib P (2020). DABA MNPs: A new and efficient magnetic bifunctional nanocatalyst for the green synthesis of biologically active pyrano[2,3-c]pyrazole and benzylpyrazolyl coumarin derivatives. New J. Chem..

[8] Babaei E, Mirjalili BBF (2019). An expedient and eco-friendly approach for multicomponent synthesis of dihydropyrano[2,3-c]pyrazoles using nano-Al_2_O_3_/BF_3_/Fe_3_O_4_ as reusable catalyst. Inorg. Nano-Met. Chem..

[9] Dadaei M, Naeimi H (2020). An environment-friendly method for green synthesis of pyranopyrazole derivatives catalyzed by CoCuFe_2_O_4_ magnetic nanocrystals under solvent-free conditions. Polycycl. Aromat. Compd..

[10] Ali E, Naimi-Jamal MR, Ghahramanzadeh R (2019). One-pot multicomponent synthesis of pyrano[2,3 c]pyrazole derivatives using CMCSO_3_H as a green catalyst. ChemistrySelect.

[11] Eivazzadeh-Keihan R (2020). Fe_3_O_4_/GO@melamine-ZnO nanocomposite: A promising versatile tool for organic catalysis and electrical capacitance. Colloids Surf. A Physicochem. Eng. Asp..

[12] Paul S, Pradhan K, Ghosh S, De SK, Das AR (2014). Uncapped SnO_2_ quantum dot catalyzed cascade assembling of four components: A rapid and green approach to the pyrano[2,3-c]pyrazole and spiro-2-oxindole derivatives. Tetrahedron.

[13] Bihani M, Bora PP, Bez G, Askari H (2013). Amberlyst A21 catalyzed chromatography-free method for multicomponent synthesis of dihydropyrano[2,3-c]pyrazoles in ethanol. ACS Sustain. Chem. Eng..

[14] Copéret C, Chabanas M, Petroff Saint-Arroman R, Basset J-M (2003). Homogeneous and heterogeneous catalysis: Bridging the gap through surface organometallic chemistry. Angew. Chem. Int. Ed..

[15] De Vries JG, Jackson SD (2012). Homogeneous and heterogeneous catalysis in industry. Catal. Sci. Technol..

[16] Ahghari MR, Soltaninejad V, Maleki A (2020). Synthesis of nickel nanoparticles by a green and convenient method as a magnetic mirror with antibacterial activities. Sci. Rep..

[17] Polshettiwar V, Varma RS (2010). Green chemistry by nano-catalysis. Green Chem..

[18] Singh SB, Tandon PK (2014). Catalysis: A brief review on nano-catalyst. JECE..

[19] Lu A-H, Salabas EL, Schüth F (2007). Magnetic nanoparticles: Synthesis, protection, functionalization, and application. Angew. Chem. Int. Ed..

[20] Polshettiwar V (2011). Magnetically recoverable nanocatalysts. Chem. Rev..

[21] Baig RBN, Varma RS (2013). Magnetically retrievable catalysts for organic synthesis. Chem. Commun..

[22] Xie W, Wan F (2018). Basic ionic liquid functionalized magnetically responsive Fe_3_O_4_@HKUST-1 composites used for biodiesel production. Fuel.

[23] Maleki A, Ghamari N, Kamalzare M (2014). Chitosan-supported Fe3O4 nanoparticles: A magnetically recyclable heterogeneous nanocatalyst for the syntheses of multifunctional benzimidazoles and benzodiazepines. RSC Adv..

[24] Rahimi J, Bahrami N, Niksefat M, Kamalzare M, Maleki A (2020). A novel biodegradable magnetic bionanocomposite based on tannic acid as a biological molecule for selective oxidation of alcohols. Solid State Sci..

[25] Kamalzare M, Bayat M, Maleki A (2020). Green and efficient three-component synthesis of 4H-pyran catalysed by CuFe_2_O_4_@starch as a magnetically recyclable bionanocatalyst. R. Soc. Open Sci..

[26] Tamoradi T, Karmakar B, Kamalzare M, Bayat M, Taheri Kal-Koshvandi A, Maleki A (2020). Synthesis of Eu(III) fabricated spinel ferrite based surface modified hybrid nanocomposite: Study of catalytic activity towards the facile synthesis of tetrahydrobenzo[b]pyrans. J. Mol. Struct..

[27] Maleki A, Kamalzare M (2014). Fe_3_O_4_@cellulose composite nanocatalyst: Preparation, characterization and application in the synthesis of benzodiazepines. Catal. Commun..

[28] Hajizadeh Z, Hassanzadeh-Afruzi F, Jelodar DF, Ahghari MR, Maleki A (2020). Cu(II) immobilized on Fe_3_O_4_@HNTs–tetrazole (CFHT) nanocomposite: Synthesis, characterization, investigation of its catalytic role for the 1,3 dipolar cycloaddition reaction, and antibacterial activity. RSC Adv..

[29] Maleki A, Kamalzare M (2014). An efficient synthesis of benzodiazepine derivatives via a one-pot, three-component reaction accelerated by a chitosan-supported superparamagnetic iron oxide nanocomposite. Tetrahedron Lett..

[30] Maleki A, Kamalzare M, Aghaei M (2015). Efficient one-pot four-component synthesis of 1,4-dihydropyridines promoted by magnetite/chitosan as a magnetically recyclable heterogeneous nanocatalyst. J. Nanostruct. Chem..

[31] Shaikh MA, Farooqui M, Abed S (2018). Novel task-specific ionic liquid [Et_2_NH(CH_2_)_2_CO_2_H][AcO] as a robust catalyst for the efficient synthesis of some pyran-annulated scaffolds under solvent-free conditions. Res. Chem Intermed..

[32] Mecadon H (2011). l-Proline as an efficient catalyst for the multicomponent synthesis of 6-amino-4-alkyl/aryl-3-methyl-2,4-dihydropyrano[2,3-c]pyrazole-5-carbonitriles in water. Tetrahedron Lett..

[33] El Mejdoubi K, Sallek B, Digua K, Chaair H, Oudadesse H (2019). Natural phosphate K09 as a new reusable catalyst for the synthesis of dihydropyrano[2,3-c]pyrazole derivatives at room temperature. Kinet. Catal..

[34] Shinde SK, Patil MU, Damate SA, Patil SS (2017). Synergetic effects of naturally sourced metal oxides in organic synthesis: A greener approach for the synthesis of pyrano[2,3-c]pyrazoles and pyrazolyl-4H-chromenes. Res. Chem. Intermed..

[35] Azizi N, Dezfooli S, Khajeh M, Hashemi MM (2013). Efficient deep eutectic solvents catalyzed synthesis of pyran and benzopyran derivatives. J. Mol. Liq..

[36] Kumar GS (2013). An efficient multicomponent synthesis of 6-amino-3-methyl-4-Aryl-2,4- dihydropyrano[2,3-c]pyrazole-5-carbonitriles. Org. Prep. Proc. Int..

[37] Reddy B, Nagarajan A (2009). Synthesis of substituted pyranopyrazoles under neat conditions via a multicomponent reaction. Synletter.

[38] Bora PP, Bihani M, Bez G (2013). Multicomponent synthesis of dihydropyrano[2,3-c]pyrazoles catalyzed by lipase from *Aspergillus niger*. J. Mol. Catal. B Enzym..

[39] Maleki A, Eskandarpour V (2019). Design and development of a new functionalized cellulose-based magnetic nanocomposite: Preparation, characterization, and catalytic application in the synthesis of diverse pyrano[2,3-c]pyrazole derivatives. J. Iran. Chem. Soc..

[40] Chavan HV, Babar SB, Hoval RU, Bandgar BP (2011). Rapid one-pot, four component synthesis of pyranopyrazoles using heteropolyacid under solvent-free condition. Bull. Korean Chem. Soc..

[41] Ghasemzadeh MA, Mirhosseini-Eshkevari B, Abdollahi-Basir MH (2018). MIL-53(Fe) metal-organic frameworks (MOFs) as an efficient and reusable catalyst for the one-pot four-component synthesis of pyrano[2,3-c]-pyrazoles. Appl. Organomet. Chem..

[42] Ghorbani-Vaghei R, Mahmoodi J, Maghbooli Y, Shahriari A (2017). A suitable one-pot synthesis of 3,4-dihydropyrano[3,2-c]chromenes using magnetic nanoparticles tag: piperidinium benzene-1,3-disulfonate ionic liquid as a novel, green, efficient and reusable catalyst in aqueous medium. Curr. Org. Synth..

[43] Moosavi-Zare AR, Zolfigol MA, Salehi-Moratab R, Noroozizadeh E (2016). Catalytic application of 1-(carboxymethyl)pyridinium iodide on the synthesis of pyranopyrazole derivatives. J. Mol. Catal. A Chem..

