# Supplementary material for: Fe3O4@chitosan-tannic acid bionanocomposite as a novel nanocatalyst for the synthesis of pyranopyrazoles
Source: Sci Rep. 2021 Oct 8;11:20021. doi: 10.1038/s41598-021-99121-2 (PMC8501051; doi:10.1038/s41598-021-99121-2)
Supplement: Supplementary file 1 — Supplementary Information. [file 41598_2021_99121_MOESM1_ESM.pdf]

Fe_3O_4@chitosan-tannic acid bionanocomposite as a novel nanocatalyst for the synthesis of pyranopyrazoles

Maryam Kamalzare [a,b],Mohammad Reza Ahghari [b],Mohammad Bayat [,a] and Ali Maleki [*,b]*

[a.] *Department of Chemistry, Faculty of Science, Imam Khomeini International University, Qazvin, Iran. E-mail: bayat_mo@yahoo.com, m.bayat@sci.ikiu.ac.ir; Tel: +98 (28)33780040*

[b.] *Catalysts and Organic Synthesis Research Laboratory, Department of Chemistry, Iran University of Science and Technology, Tehran 16846-13114, Iran. E-mail: maleki@iust.ac.ir; Fax: +98 21 73021584; Tel: +98 21 73228313*

Table of contents

Subject **Page**

Table S1. Magnetic parameters of Fe_3O_4 and Fe_3O_4@chitosan-tannic acid S2

Table S2. Optimization of the reaction conditions in the synthesis of product **(5a)**......................................S2

Figure S1. Elemental mapping of Fe_3O_4@chitosan-tannic acid bionanocomposite S3

Figure S2. Reusability of the Fe_3O_4@chitosan-tannic acid bionanocomposite S3

Figure S3. XRD patterns of the Fe_3O_4@chitosan-tannic acid bionanocomposite and recycled bionanocomposite...S4

Figure S4. EDX analysis of the recycled Fe_3O_4@chitosan-tannic acid bionanocomposite..........................S4

Figure S5. ^{1}HNMR spectrum of the product **(5g)** S5

Figure S6. ^{13}C NMR spectrum of the product **(5g)** S6

Figure S7. ^{1}HNMR spectrum of the product **(5c)** S7

Table S1. Magnetic parameters of Fe_3O_4 and Fe_3O_4@chitosan-tannic acid

Sample	Ms (emu/g)	Mr (emu/g)	Hc (Oe)
Fe_3O_4	56.07	2.12	27.03
Fe_3O_4@chitosan-tannic acid	36.54	6.1	80.02

Table S2. Optimization of the reaction conditions for the synthesis of pyranopyrazole derivatives

Entry	Solvent	Temperature	Catalyst (mg)	Yield (%)
**1**	Water	r.t.	-	Trace
**2**	Ethanol	r.t.	-	Trace
**3**	Ethanol	r.t.	10	69
**4**	Ethanol	r.t.	20	94
**5**	Ethanol	r.t.	30	93
**6**	Ethanol	Reflux	10	89

**7**	Ethanol	Reflux	20	90
**8**	Acetonitrile	r.t.	20	78

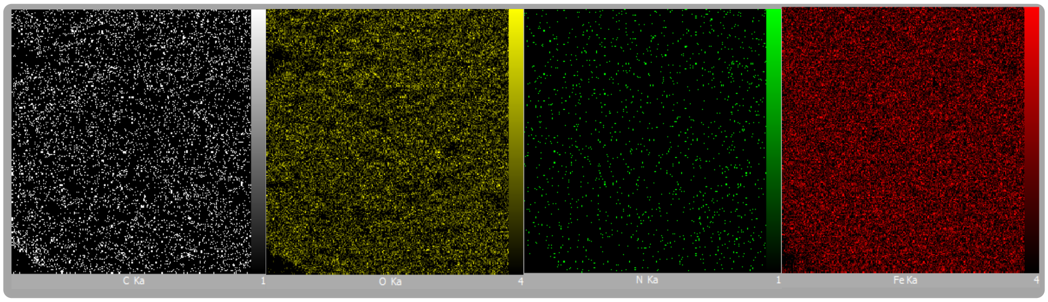

Figure S1. Elemental mapping of Fe_3O_4@chitosan-tannic acid bionanocomposite

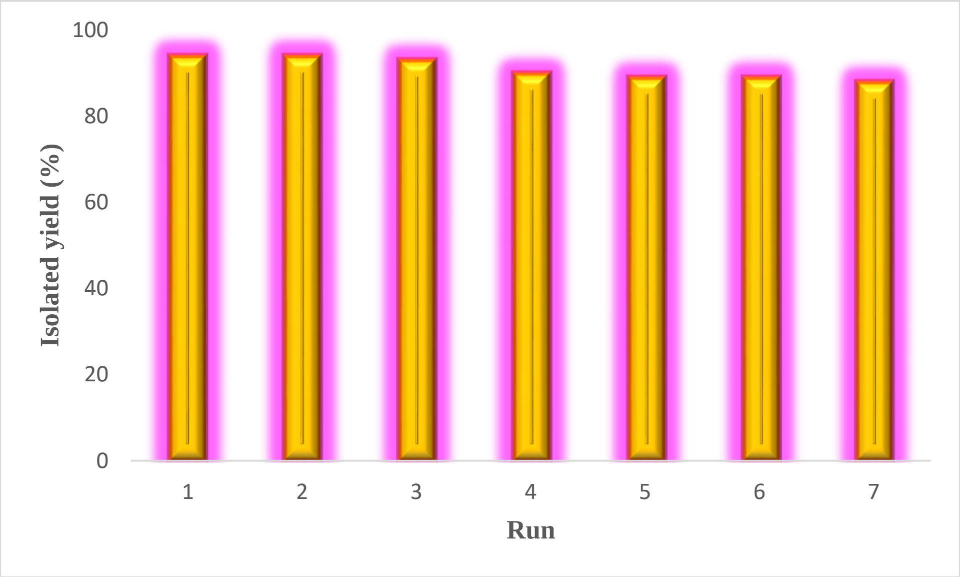

Figure S2. Reusability of the Fe_3O_4@chitosan-tannic acid bionanocomposite

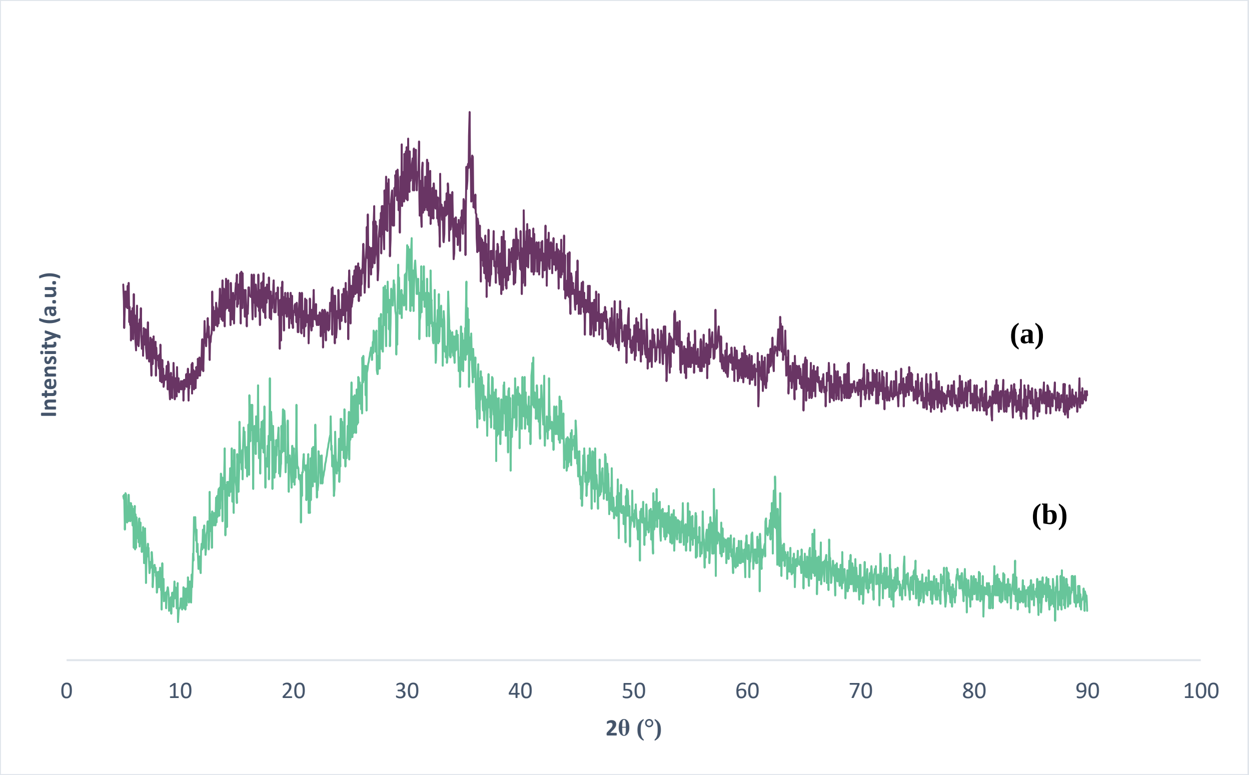

Figure S3. XRD patterns of (**a**) Fe_3O_4@chitosan-tannic acid bionanocomposite; and (**b**) recycled bionanocomposite

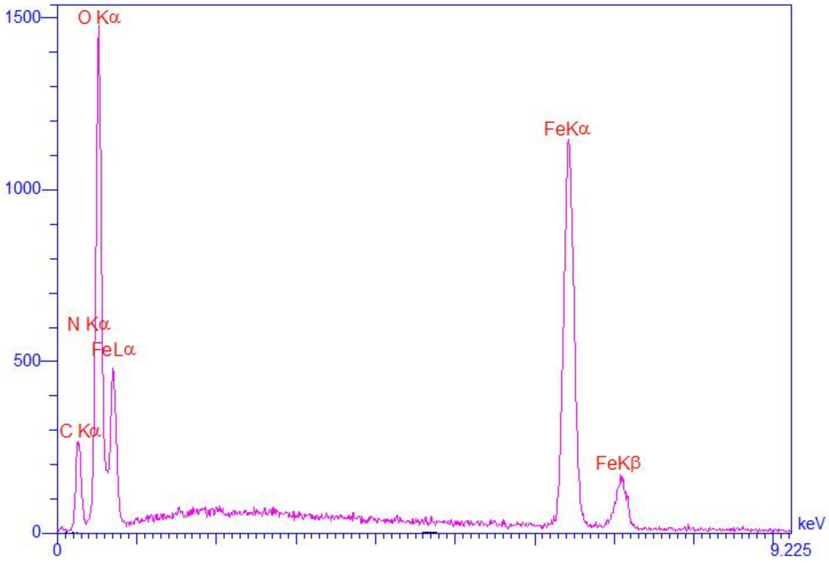

Figure S4. EDX analysis of recycled Fe_3O_4@chitosan-tannic acid bionanocomposite

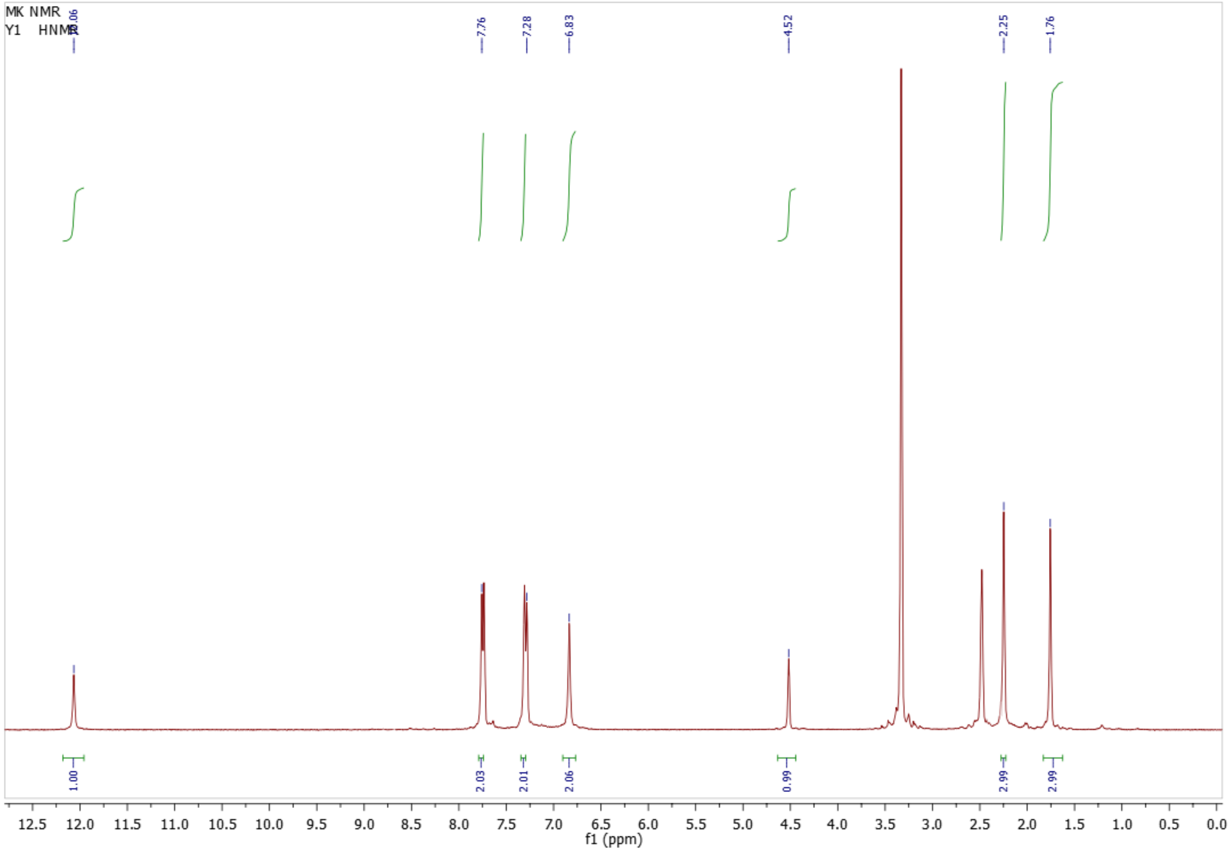

Figure S5. ^{1}H NMR spectrum of the product **5g**

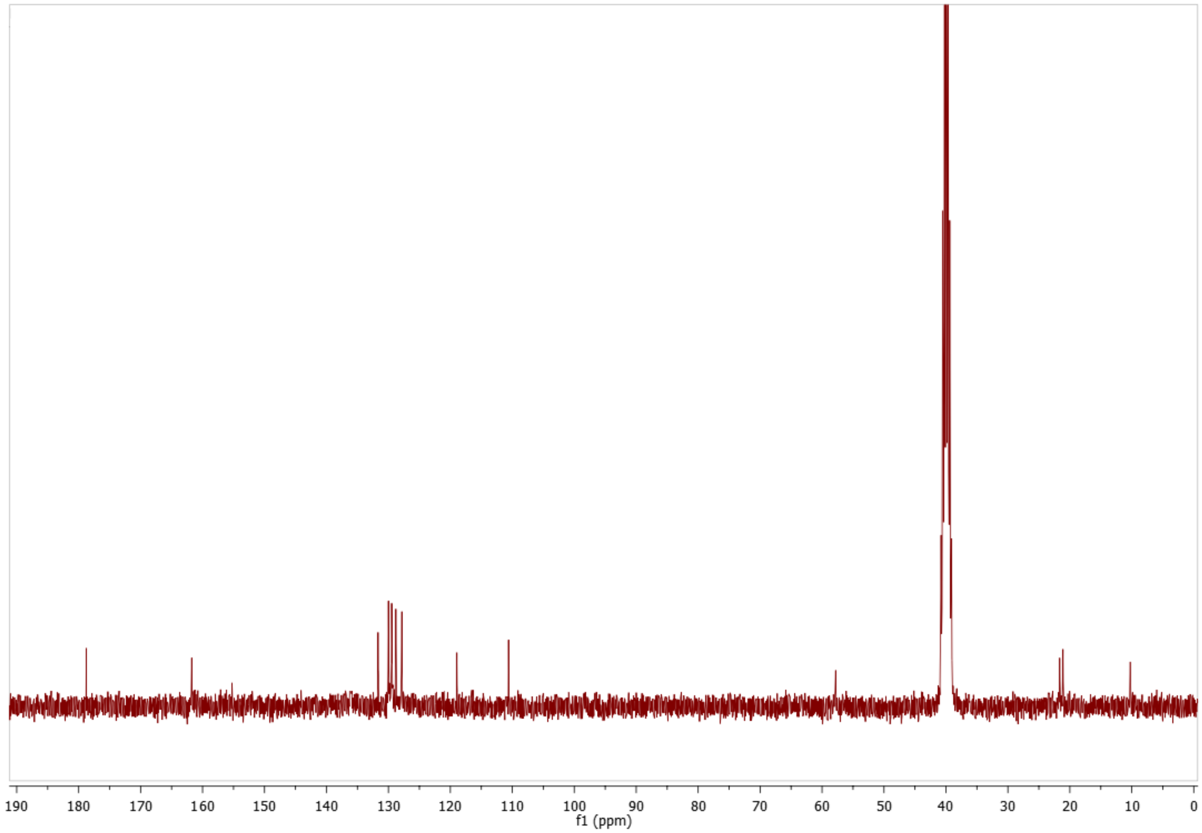

Figure S6. ^{13}C NMR spectrum of the product **5g**

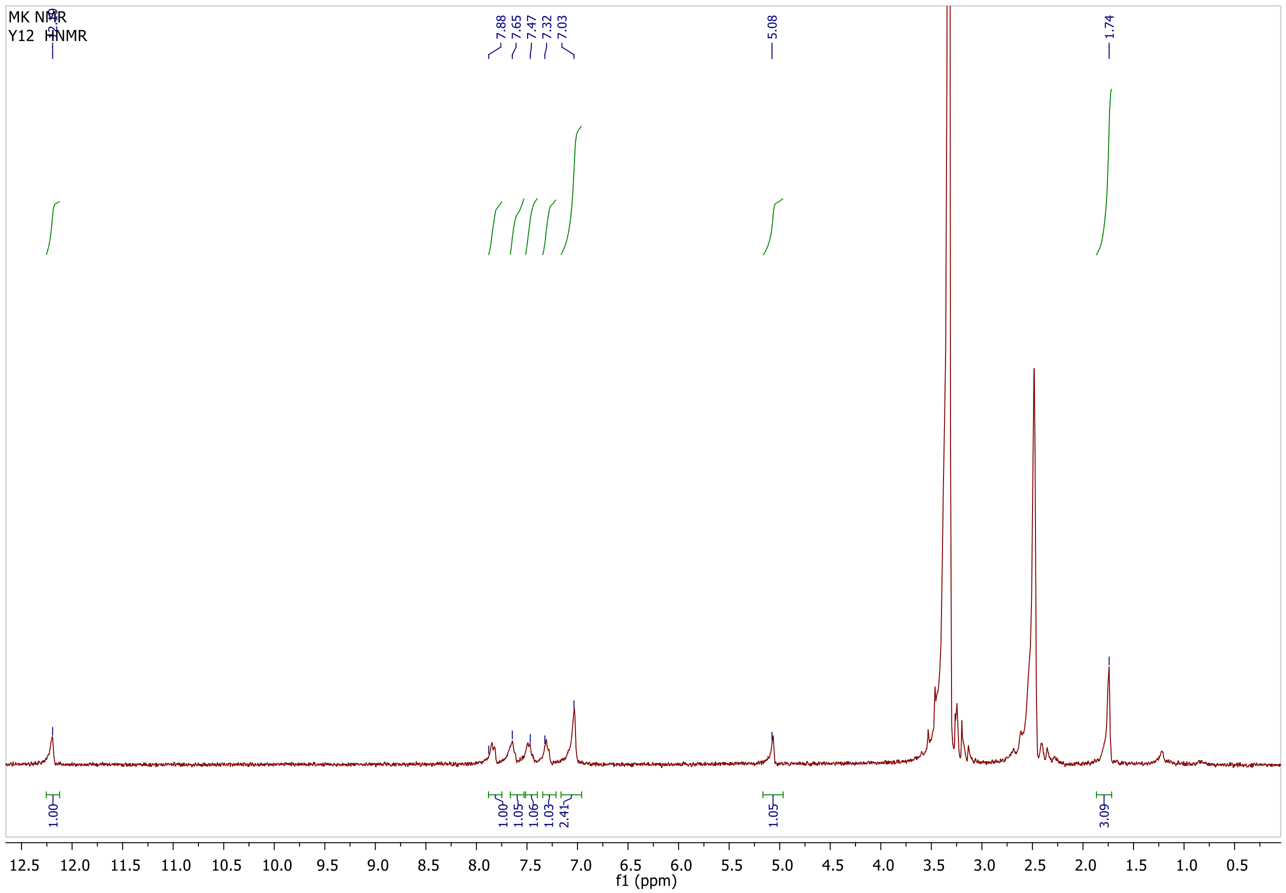

Figure S7. ^{1}H NMR spectrum of the product **5c**
